# Homologous Series of Polyaniline Derivatives Block Copolymers with Amphiphilic and Semiconducting Properties

**DOI:** 10.3390/polym14112149

**Published:** 2022-05-25

**Authors:** Ana-Maria Solonaru, Mihai Asandulesa, Andrei Honciuc

**Affiliations:** Electroactive Polymers and Plasmochemistry Laboratory, “Petru Poni” Institute of Macromolecular Chemistry, Aleea Gr. Ghica Voda 41A, 700487 Iasi, Romania; solonaru.anamaria@icmpp.ro (A.-M.S.); asandulesa.mihai@icmpp.ro (M.A.)

**Keywords:** polyaniline, semiconducting polymers, amphiphilic polymers, interfacial activity

## Abstract

Semiconducting polymers with amphiphilic properties can play an increasing role in future organic and unimolecular electronic devices, especially due to their excellent processability and ease of self-assembly into thin films, but they could also be used as intermediate layers to improve electron transport in metal-organic junctions. In this work, we synthesized a homologous series of amphiphiles by copolymerization of aniline with aniline-*N*-propanesulfonic acid. The polymerization was first initiated with aniline, and the latter monomer was added at different time intervals: 2, 10, 20, 30, 40, and 60 min, spaced from the time of initiation. Thus, the poly(aniline-co-aniline-*N*-propanesulfonic acid) (PANi-co-PANs) homologous series of copolymers obtained had the same length of the water soluble PANs chain, and a variable length of the water insoluble PANi chain. We demonstrated that there is a strong structure–activity relationship in the homologous series of PANi-co-PANs copolymers, evidenced in the tensiometry and wettability studies, as well as in-depth conductivity with frequency and temperature investigations. We observed a gradual change in solubility, interfacial activity, and conductivity in the homologous series of amphiphiles within the boundaries set by the electrically insulating, hydrophilic PANs chain and the semiconducting, hydrophobic PANi chains; representing a viable platform toward designing polymers with tunable conductivity.

## 1. Introduction

Multifunctionality in polymer materials stems from a manifold of combinations between a measurable property in the bulk material or powder (conductivity, magnetism, elasticity), the electronic structure (conjugation), and arrangements of blocks in the polymer backbone (amphiphilicity, pseudo-amphiphilicity, etc.). Semiconducting polymers are a large class of polymer materials with increasing importance for electronics and organic electronics. Among these, polyaniline can be named “the queen” among conducting polymers, due to very large number of publications in the specialty literature [1,2,3,4], and on other hand due to its attractive properties, such as a high electrical conductivity [5,6,7,8], in the order of 10 S/cm, depending on the preparation method in the presence of various organic or inorganic acids, such phosphoric, picric, camphorsulphonic, or hydrochloric acid [9]; ease of preparation; stable redox activity; and environmental stability [10,11,12].

One of the major known drawbacks of polyaniline is its poor processability in solvents, and especially in aqueous solvents. Among the strategies used to alleviate this problem and improve its solubility in water is to introduce polar functional groups to the benzene ring or to the nitrogen atom in the aniline monomer, to obtain modified polyanilines. For example, water-soluble polyaniline-*N*-propanesulfonic acid can be obtained by ring opening-addition reaction of 1,3-propanesultone to aniline monomer, which can be polymerized by oxidative addition [13]. Other ways to obtain soluble sulfonated polyaniline are the treatment of emeraldine base of PANi with 1,3-propanesultone or 1,4 butanesultone in the presence of sodium hydride [14,15], as well as the direct ring-sulfonation of the emeraldine base form with fuming sulfuric acid, when –SO_3_H groups are introduced at the aromatic ring [16]. In addition, a good water solubility has a series of alkyl polyanilines substituted at the *N* atom, with groups such as methyl, ethyl, butyl, and ethyl alcohol, and synthesized by enzymatic polymerization in the presence of sodium polystyrene sulfonate [17] and polyaniline bearing phosphonic acid obtained via oxidative polymerization of 2-methoxyaniline-5-phosphonic acid [18,19]. While this strategy proved to work well, the main effect of *N-*substitution is that the overall conductivity can dramatically decrease, due to the decrease in the conjugation length [20]. In this work we address this issue of improving the water solubility of a polyaniline semiconductor by designing a semiconducting polymer consisting of a block copolymer of polyaniline and a block of more polar polyaniline derivatives, consisting of propyl sulfone moieties grafted onto the nitrogen atom. In doing so, an additional property arises, due to the different polarity between the two blocks, namely amphiphilicity. By tuning the reaction conditions, in such a way that the more hydrophilic polyaniline-*N*-propanesulfonic acid (PANs) block remains constant in length and the more hydrophobic polyaniline (PANi) block increasingly becomes longer, we can vary both the conductivity of the obtained PANi-co-PANs copolymer but also its amphiphilicity. In doing so, a homologous series of multifunctional polymers of polyanilines are obtained, which combine both amphiphilic and semiconducting properties, which vary gradually in the homologous series. This new class of amphiphilic semiconducting polymers could greatly expand the application domain of semiconducting polymers into the application realm of classic surfactants, such as emulsification, emulsion polymerization, and interfacial adsorption, and endow surfaces and interfaces with conductive properties. Furthermore, amphiphilic semiconducting molecules are extremely important for potential use as active components in unimolecular and molecular electronic devices.

## 2. Experimental

### 2.1. Materials

Ammonium persulfate (APS) (>99%), 1,3-propane sultone (98%), acetonitrile (anhydrous, 99.8%), PTFE sheet (3.18 mm, (0.125 mm) thick), polypropylene sheet (6.36 mm (0.25 in) thick), polyethylene sheet (high density, 12.7 mm (0.5 in) thick), and hydrochloric acid (37%) were purchased from Sigma-Aldrich (Merck KGaA, Darmstadt, Germany). Aniline (AN) (from Reactivul Bucuresti, Bucharest, Romania) was double distilled under reduced pressure just before use and stored in the dark in nitrogen. Freshly distilled water was used to prepare all aqueous solutions. The synthesis of aniline-*N*-propanesulfonic acid (AnPS) was previously reported [13].

### 2.2. Synthesis of Amphiphilic Polyaniline Block Copolymers PANi-co-PANs

The synthesis of conducting amphiphilic polyaniline block copolymers PANi-co-PANs was performed by chemical oxidative polymerization as follows: 10 µL aniline and 3 mL 1 M HCl solution were added in six 50-mL flasks and cooled at 0–5 °C in an ice bath, then 36 mg APS dissolved in 1 mL 1 M HCl solution (molar ration between aniline and oxidant = 1/1.5) was added dropwise over a period of 5 min under stirring (Solution A). Then, when the color of Solution A turned green (after approximately 15–20 min), 230 mg AnPS dissolved in 3 mL 1 M HCl solution was added. To vary the chain length of PANi, the AnPS was added at different times intervals: after 2, 10, 20, 30, 40, and 60 min, spaced from the moment the Solution A turned green. After 40 min and longer, an insoluble fraction separates out from the Solution A, signaling that longer chain PANi has separated out from the reaction mixture. At 40 min, Solution A, after separation of the insoluble PANi fraction, remains strongly green colored, while at 60 min it is only pale green, indicating that at longer times, more PANi separates out of the reaction mixture. The reaction vessel was further stirred for 8 h at 0–5 °C in an ice bath. The copolymer solutions were dialyzed for 8 h, during which time the water was changed several times. The solution was evaporated and dried at 50 °C. Yields ranged from 85% to 90%.

### 2.3. Characterization Methods

#### 2.3.1. FTIR, NMR and UV-Vis Spectroscopy

FTIR spectra were recorded by depositing dried powder sample on the ATR crystal using a DIGILAB-FTS 2000 spectrometer (Bruker, Karlsruhe, Germany) in the range of 400 to 4000 cm^−1^. ^1^H-NMR spectra were recorded using a NMR spectrometer, 400 MHz, for liquids (type Bruker Avance Neo 400, Rheinsteitten, Germany) in deuterated water (D_2_O). The UV–Visspectra of all amphiphilic copolymers were measured in aqueous solution with a concentration 10 mg/mL using an UV–Vis SPECORD 200 Analytik Jena spectrometer (Analytik Jena AG, Jena, Germany)

#### 2.3.2. Surface Tension Measurement

Surface tension (ST) measurements were carried out via pendant-drop analysis method. The experiments were done using a DataPhysics OCA 15 (DataPhysics GmbH, Filderstadt, Germany) contact angle goniometer, equipped with an automatic dosing system. For concentration surface tension measurements, we prepared a stock solution for each copolymer with a concentration of 100 mg/mL and then adjusted it by adding 0.5 mL each time, to obtain a new concentration (100 mg/1.5 mL, 100 mg/2 mL, 100 mg/2.5 mL, 100 mg/3 mL).

#### 2.3.3. Contact Angle Measurements

Contact angles of aqueous solution of each amphiphilic copolymers were measured using a DataPhysics OCA 15 (DataPhysics GmbH, Filderstadt, Germany) optical contact angle goniometer. The contact angle measurements for all samples were performed at a concentration of 100 mg/mL. The contact angle was measured immediately after generating the sessile drop on the substrate. The substrates used were polyethylene (PE), polypropylene (PP), polytetrafluoroethylene (PTFE), quarts, and regular microscope glass. All substrates were washed and cleaned with distilled water and isopropanol, then left at room temperature to dry.

#### 2.3.4. Conductivity Measurements

Prior to electrical measurements, all the samples were dried at 50 °C, for 24 h, to eliminate the solvent residues. Then, the powder samples were compressed at room temperature at a pressure of 10 tons into pellets of 13 mm in diameter and around 0.03 cm in thickness.

The conductivity of amphiphilic polyaniline copolymers (PANi-co-PANs) and PANs was recorded with a Novocontrol Concept 40 Broadband Dielectric Spectrometer (Novocontrol GmbH, Montabaur, Germany) device equipped with an Alpha-A High Performance Frequency Analyzer. The conductivity was evaluated in two different manners: alternating current (ac) regime and direct current (dc) regime. In the ac-regime, the complex conductivity function σ*(ƒ,T) = σ’(ƒ,T) + iσ”(ƒ,T) was recorded in a wide range of frequency, between 0.1 Hz and 10 MHz. In order to prevent the non-linear effects that can appear at the interfaces between the material under study and the electrodes employed for dielectric measurements, the sinusoidal voltage of the frequency sweeps was set at 0.2 V. In the dc-regime, the dc voltage applied to the sample was set between −0.15 V and 0.15 V, and the measured value of the sample dc current was further collected. The resistance (R_dc_) was evaluated from the slope of current-voltage (I-V) sweeps, and the electrical conductivity (σ_dc_) was finally calculated with the equation: σ_dc_ = d/R_dc_·A, where d (≈0.3 mm) is the thickness and A (≈132.67 mm^2^) represents the surface area of the measured pellet. Both the frequency and the I-V scans were taken under isothermal conditions, at temperatures between 0 °C and 60 °C. The ramping of temperature was assisted by a Quatro Cryosystem device which provides 0.1 °C stability and high reproducibility. Moreover, the temperature was controlled by a flow of dry nitrogen atmosphere, excluding, thus, the moisture from environment.

Since the PANi reference is a polymer with high conductivity values, its electrical properties were separately measured with a standard four-point probe technique, operating at room temperature. The I-V sweeps were measured with a Keithley 2428. The electrical conductivity of PANi was evaluated similarly to that from dielectric measurements working in the dc-regime.

## 3. Results and Discussions

### 3.1. Synthesis of a Homologous Series of PANi-co-PANs Amphiphiles

**Scheme 1:**
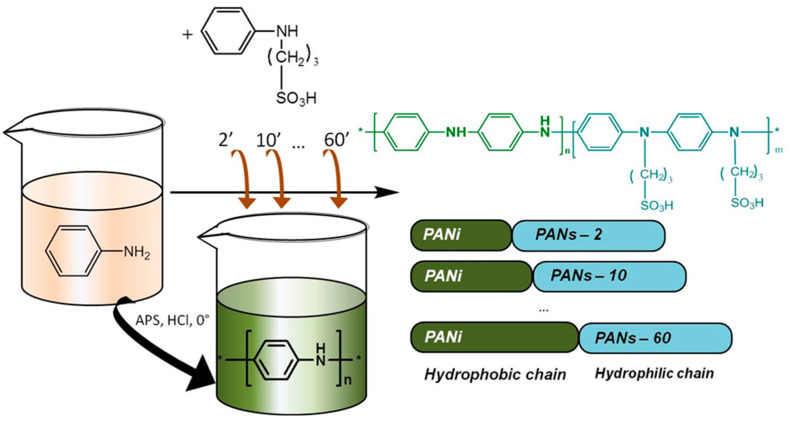
The reaction for preparation of PANi-co-PANs amphiphilic copolymers. The addition of aniline *N*-propane sulfonic acid monomer was timed from the moment the polymerization vessel containing polyaniline turned green.

The conductive amphiphilic copolymers based only polyanilines were successfully prepared by in situ oxidative polymerization in two steps. The first step consisted of the beginning of the initiation and polymerization of aniline, and the second step consisted of adding the AnPS at different time intervals, spaced from the moment of the appearance of the green color in the first step, at: 2, 10, 20, 30, 40, and 60 min, as indicated in the Scheme 1. After adding AnPS, the polymerization reaction continued for another 8 h, for all samples. We obtained, in this way, a homologous series of PANi-co-PANs polymers: PANi-co-PANs-2, PANi-co-PANs-10, PANi-co-PANs-20, PANi-co-PANs-30, PANi-co-PANs-40, and PANi-co-PANs-60; the number indicates the time interval of the addition of the monomer in the second step. The presence of sulfonic acid groups in the polymer structure led to the formation of water soluble copolymers at the beginning of the homologous series: PANi-co-PANs-2, PANi-co-PANs-10, PANi-co-PANs-20; partially soluble: PANi-co-PANs-30; and water insoluble: PANi-co-PANs-40 and PANi-co-PANs-60. This behavior indicates the gradual increase in the hydrophobic PANi chain within the homologous series, from PANi-co-PANs-2 to PANi-co-PANs-60. When AnPS was added at a time interval of 40 min or longer an insoluble PANi fraction begins forming, yet we allowed continuation of the polymerization of AnPS for another 8 h, the same as for all the other samples. The fact that the obtained copolymers PANi-co-PANs-40 and PANi-co-PANs-60 are insoluble in water can be explained in that PANs is too short to aid with the solubility of the copolymer. While we were tempted to treat PANi-co-PANs-40 and PANi-co-PANs-60 as a purely PANi and PANs composite mixture, this is not the case, because PANi and PANs are clearly chemically bonded, otherwise in a physical mixture PANs would be soluble in water, yielding a green solution. In this case, however, the PANi-co-PANs-40 and PANi-co-PANs-60 were completely insoluble in water. PANi-co-PANs-40 and PANi-co-PANs-60 were partially soluble in ethanol, isopropanol, DMSO, and acetone, see Figure S1.

### 3.2. FTIR Spectroscopy

**Figure 1:**
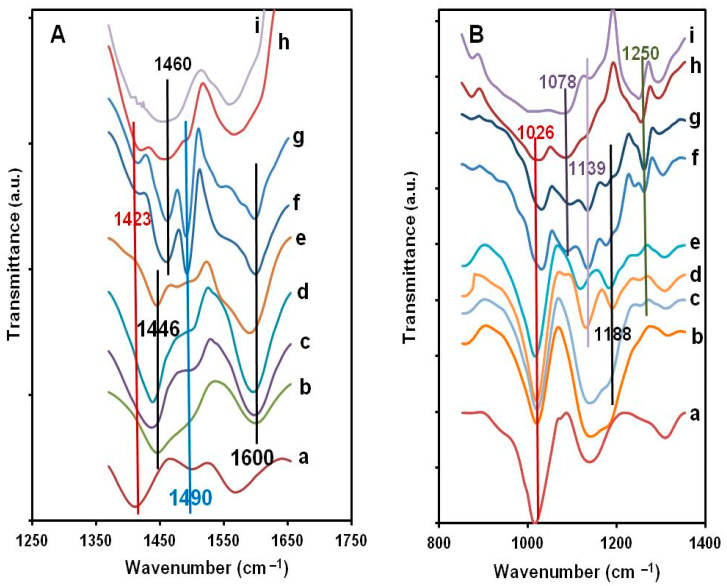
FTIR spectra of amphiphile polyanilines copolymers PANi-co-PANs, PANs, PANi, and physical mixture PANi-PANs. (**A**) for the region 800–1400 cm^−1^ and (**B**) for region 1350–1650 cm^−1^. Each spectrum was normalized by the highest intensity peak and offset. (a) PANs, (b) PANi-co-PANs-2, (c) PANi-co-PANs-10, (d) PANi-co-PANs-20, (e) PANi-co-PANs-30, (f) PANi-co-PANs-40, (g) PANi-co-PANs-60, (h) physical mixture PANi- PANs, and (i) PANi.

The FTIR spectra of PANi-co-PANs amphiphilic copolymers, PANs, PANi, and physical mixture PANi-PANs (1:1) are shown in Figure 1 and Figure S2. From the literature, it is known that the characteristic peaks for polyaniline are located at 1564–1571 cm^−1^ (C=C stretching of the quinoid rings), 1485–1477 cm^−1^ (C=C stretching of benzenoid rings), 1294–1300 cm^−1^ (C–N stretching vibrations), and 850–700 cm^−1^ (v C–H out of plane bending of 1,4 rings, and respectively, 1,2 rings) [21]. The PANi synthesized by us had these bands. The characteristic bands of PANs are localized at 1562 cm^−1^ (C=C stretch of quinoid ring), 1423 cm^−1^ (benzenoid ring), 1313 cm^−1^ (C–N stretching in the benzenoid and quinoid imine), 1031 cm^−1^, and 1153 cm^−1^ (asymmetric and symmetric sulfonic groups O=S=O stretching vibrations) [14,22], Figure S2. A similar behavior is generally observed for all amphiphilic copolymers based on polyanilines. In all spectra of copolymers, the bands associated to quinoid and benzenoid rings appeared at 1585 and 1442 cm^−1^ for PANi-co-PANs-2, 1589 and 1435 cm^−1^ for PANi-co-PANs-10, 1590 and 1450 cm^−1^ for PANi-co-PANs-20, 1593 and 1450 cm^−1^ for PANi-co-PANs-30, and, respectively, for PANi-co-PANs-40 and PANi-co-PANs-60 at 1600 and 1460 cm^−1^, as shown in Figure 1A and Figure S2. These bands are specific also to PANi and PANs, but only in copolymers are they shifted to the right, to larger wavenumbers. Moreover, the bands assigned to the asymmetric and symmetric O=S=O stretching vibration, Figure 1B and Figure S2, can be observed at 1157 and 1026 cm^−1^ to copolymers PANi-co-PANs-2, at 1145–1026 cm^−1^ to PANi-co-PANs-10, at 1188 and 1026 cm^−1^ for copolymers PANi-co-PANs-20, PANi-co-PANs-30, PANi-co-PANs-40, and PANi-co-PANs-60. In the work of Zhang et al. [23], the broad band between 1100 and 1200 cm^−1^ for PANs, PANi-co-PANs-2, and PANi-co-PANs-10 was assigned to the overlapping of C–N stretching and –SO_3_H bands. For PANi-co-PANs-20, PANi-co-PANs-30, PANi-co-PANs-40, and PANi-co-PANs-60 copolymers, with the increase the hydrophobic chain, this band is divided into two specific bands. The bands at 1139 cm^−1^ and, respectively, 1118 cm^−1^ are characteristic of C–N stretching vibration, while the peaks at 1188 cm^−1^ are due to the –SO_3_H band. The band at 1139 cm^−1^ was considered by MacDiarmid et al. [24] as a peak specific to conductive PANi, as a measure of the delocalization of electrons of PANi. The band assigned to C–N stretching of a secondary amine [25] appeared at 1307, 1303, 1300, and 1292 cm^−1^, see Figure S2. All copolymers presented the bands due to C–H out of plane bending of 1,4 rings and 1,2 rings at 856 and 875 cm^−1^ and, respectively, 752 and 788 cm^−1^ characteristics of PANi, Figure S2. To elucidate whether the PANi-co-PANs-40 and PANi-co-PANs-60 are copolymers or physical mixtures, we prepared a PANi-PANs (*w*/*w*, 1/1) polymer mixture. With further analysis of the FTIR spectra from Figure 1 of PANi-co-PANs-40 and PANi-co-PANs-60, with respect to reference samples PANi, PANs and the physical mixture PANi-PANs we observed the following: (i) the bands at 1250 cm^−1^, 1139 cm^−1^ and 1078 cm^−1^ are specific to PANi, and at 1026 cm^−1^ specific to PANs; (ii) in Figure 1A, the bands specific to PANi appear at 1460 cm^−1^, and the bands specific to PANs at appear at 1423 cm^−1^ and 1490 cm^−1^, and all these bands also appear in the physical mixture PANi-PANs; (iii) the specific bands of PANi-co-PANs-40 and PANi-co-PANs-60, appear at 1188 cm^−1^, 1250 cm^−1^, and 1600 cm^−1^ are specific only to copolymers. These results indicate that PANi-co-PANs-40 and PANi-co-PANs-60 are copolymers, and not composites.

### 3.3. ^1^H-NMR Spectroscopy

The ^1^H-NMR spectra of copolymers were recorded in D_2_O. The NMR figures for copolymers PANi-co-PANs-2–PANi-co-PANs-30 are shown in Figure S3. The signals that appeared between 2.0–3.6 ppm belong to aliphatic protons of the propane sulfonic substituent in PANs, while the aromatic protons are localized between 6.8–7.6 ppm. Since the synthesis was performed using APS as oxidant, the signals specific to ammonium cations (NH_4_^+^) can also appear in the NMR spectrum in the aromatic region. This explanations is supported by the research of Wang et al. [26]. Even if the copolymers were purified by dialysis, we could not fully control the presence of APS, because they are hydrophilic and soluble. To investigate the difference between copolymers, we integrated the multiplet signal belonging to the group –N–C–CH_2_–C– (ʃ_Alifatic_) from the propansulfonic rest located at 2.05 ppm and one of the signals given by aromatic protons in phenyl rings of polyanilines (ʃ_Aromatic_). The value of the ratios of the ʃ_Aliphatic_/ʃ_Aromatic_ integrals decreased in the homologous series, from PANi-co-PANs-2 to PANi-co-PANs-30, see Table S1. This confirmed that the PANi segment increases in the homologous series from PANi-co-PANs-2 to PANi-co-PANs-30 by almost a factor of three.

### 3.4. UV-Vis Spectroscopy

**Figure 2:**
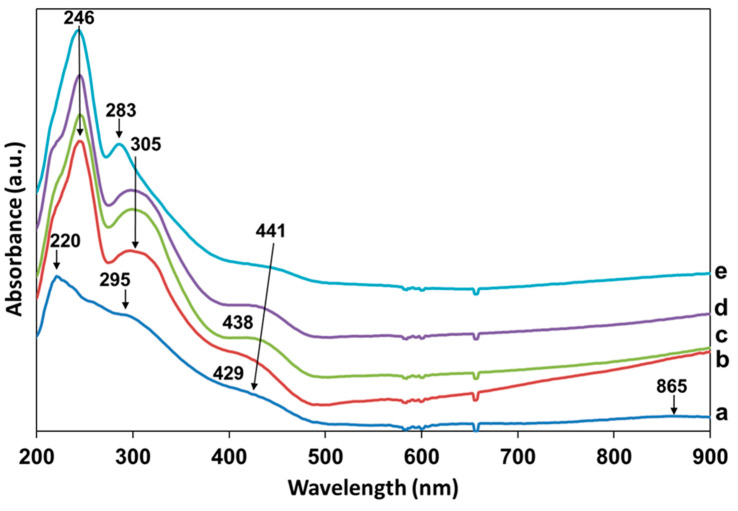
UV-Vis spectra of amphiphile polyanilines copolymers PANi-co-PANs and PANs in water. Each spectrum was normalized by the highest intensity peak and offset. (a) PANs, (b) PANi-co-PANs-2, (c) PANi-co-PANs-10, (d) PANi-co-PANs-20, and (e) PANi-co-PANs-30.

Figure 2 shows the ultraviolet-visible spectra of the amphiphilic PANi-co-PANs-2 to PANi-co-PANs-30 copolymers, and PANs in deionized water. It is known from the literature that the absorption spectrum of PANi-emerlaldine base exhibits two absorption bands between 315–345 nm and 590–650 nm, their position depending on the synthesis and processing methods. The first band is associated to the π–π* transition, and the second to the excitation of the amine segment of the polymer chain. The other form of PANi, emeraldine salt shows three absorption peaks between 325–360 nm, associated with π–π* electron transition within benzenoid rings, the second at 400–440 nm is due to polaron to π* transition, and the last one at 780–825 nm is due to π-to-polaron transition [27,28]. Furthermore, PANs spectrum exhibits three absorption bands, with the maximum at 220 nm, 296 nm, 429 nm, and a shoulder at 865 nm. The band with the maximum at 220 nm is attributed to the characteristic absorption of the benzene ring substituted with auxochrome groups and can be associated to a the n-π* transition, due to the non-bonding electrons present in the–N–(CH_2_)_3_–SO_3_H [29,30]; the band at 295 nm is due to the π-π* transition of the benzenoid ring and the peaks at 429 nm, and the shoulder at 865 is assigned to the polarons band transitions, which suggest that the polymer is in the form emeraldine salt, the conductive form. For the amphiphilic copolymers PANi-co-PANs-2 to PANi-co-PANs-30 three peaks were observed. The first absorption band in copolymers is localized at 246 nm, due to absorption of the n-π* transition exhibiting a bathochromic shifted compared to PANs, and it should be highlighted that this transition is not observed in the spectrum of PANi. The second band in copolymers at 305 nm for PANi-co-PANs-2 to PANi-co-PANs-20 and 283 nm for PANi-co-PANs-30 correspond to the π-π* transition, whereas the third band between 431–438 nm region are assigned to the formation of polaronic bands. It can be stated that the copolymer with the longest hydrophobic chain, PANi-co-PANs-30, has the third band shifted to red compared to PANs (429 nm) and is approaching the same wavelength as PANi (441 nm).

### 3.5. Interfacial Activity of PANi-co-PANs

**Figure 3:**
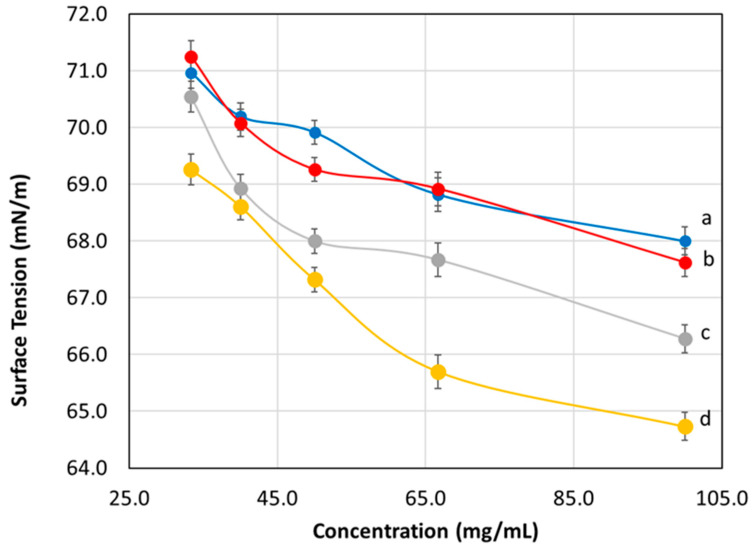
Surface tension vs. concentration plots for the homologous series of amphiphilic copolymers: (a) PANi-co-PANs-2, (b) PANi-co-PANs-5, (c) PANi-co-PANs-10, and (d) PANi-co-PANs-20.

The interfacial activity of the PANi-co-PANs amphiphilic polymers was investigated by measuring the surface tension using the pendant drop and contour shape analysis method. The surface tension vs. concentration data for the water-soluble amphiphilic polymers, PANi-co-PANs-2 to PANi-co-PANs-20, are presented in Figure 3. From the surface tension vs. concentration curves, in Figure 3, the PANi-co-PANs-20, with the longest hydrophobic PANi chain, exhibits a most prominent decrease in surface tension with the increase in concentration from ca. 25 mg/mL to 100 mg/mL, followed by PANi-co-PANs-10, PANi-co-PANs-5, and PANi-co-PANs-2, in the same order as the decreasing length of the PANi hydrophobic chain. This suggests that PANi-co-PANs-20 is the most effective at lowering the water surface tension, owing to having the longest hydrophobic chain. Here, we note that the gradual increase in interfacial activity of the PANi-co-PANs polymer with the increase in the hydrophobic PANi chain is direct evidence of the link that exists between the structure of the polymer and its interfacial activity. While a surface tension plateau was not reached for any of the amphiphiles investigated, i.e., the surface tension vs. concentration curve appears to continuously drop even at 100 mg/mL, a clear break in the curve can be noted only for PANi-co-PANs-20 at around 60 mg/mL, which we believe to correspond to the critical micelle concentration (CMC). Furthermore, judging from the lowest values of the surface tension achieved, ≈64 mN/m, it can be concluded that these semiconducting amphiphiles have a moderate to low interfacial activity.

### 3.6. Contact Angle of PANi-co-PANs and PANs Aqueous Solutions on Various Substrates

**Figure 4:**
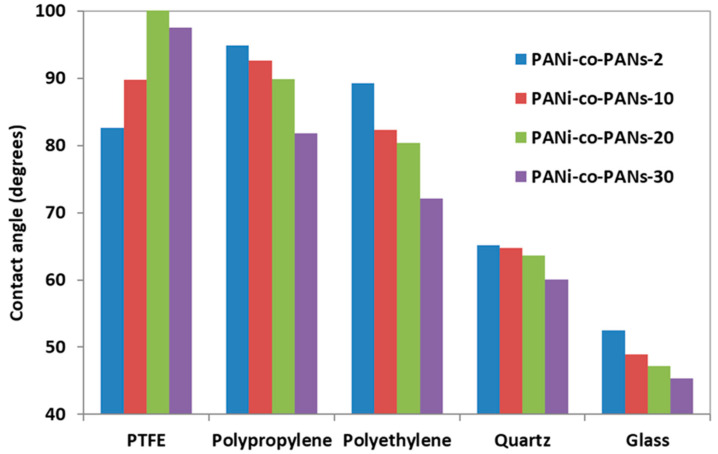
Contact angles of the of the homologous series of amphiphilic semiconducting polyaniline copolymers on various substrates.

To further demonstrate the amphiphilic nature of the semiconductive polyaniline copolymers obtained and their interfacial activity, we measured the contact angle of an aqueous solution of 10 mg/mL on five different substrates: polyethylene (PE), polypropylene (PP), polytetrafluoroethylene (PTFE), quartz, and glass. The contact angles of the homologous series of PANi-co-PANs copolymers were measured using the sessile drop method, with an optical contact angle goniometer, see Figure 4. The contact angles of water for these substrates are shown in Figure S4 and are also reported in literature [31,32]. For comparison, we also measured the contact angle of the hydrophilic PANs at the same concentration, and these values are reported in Figure 4. Several trends can be noted from the data presented in Figure 4.

First, for hydrophobic surfaces, PE and PP, and hydrophilic substrates Quartz and glass, the contact angles of the aqueous solutions of the homologous series of polyaniline copolymers decreased with the increase in the PANi hydrophobic chain, from PANi-co-PANs-2 to PANi-co-PANs-20. This evolution seems to support the fact that the PANi-co-PANs-20 lowers the surface tension of water the most, i.e., highest interfacial activity. However, we also note that the exception is the PTFE substrate, which is both oleophobic and hydrophobic, and the contact angle values increased in the homologous series of amphiphilic copolymers from PANi-co-PANs-2 to PANi-co-PANs-20. The increase in the contact angle value for PTFE, and decrease of the contact angle values monotonically in the homologous series of PANi-co-PANs, with the increase in the hydrophobic chain length, suggests that the lowering of surface tension is not the only mechanism by which the amphiphilic copolymers lower the contact angle value; and that, in fact, adsorption at, or ahead of, the three-phase line may also be involved [33].

Second, the values of the contact angles decreased with increase in the surface energy of the substrate, from PTFE ($\gamma_{PTFE}^{total}=14$, $\gamma_{PTFE}^{polar}=1.5$) [34], PE ($\gamma_{PE}^{total}=33.1$) [34], PP ($\gamma_{PP}^{total}=30.1$), quartz ($\gamma_{quartz}^{total}=213.4$) [35], glass ($\gamma_{glass}^{total}=3500-5300$) [36], which is an increase in the ability of the substrate’s surface to interact via physical forces.

The evolution of the contact angle observed in the two trends seems to indicate that lowering the surface tension of the water by the PANi-co-PANs homologous series, but also some interfacial adsorption at the solid–liquid interface, is responsible for the wettability behavior observed.

### 3.7. Conductivity Studies of the Homologous Series of PANi-co-PANs

**Figure 5:**
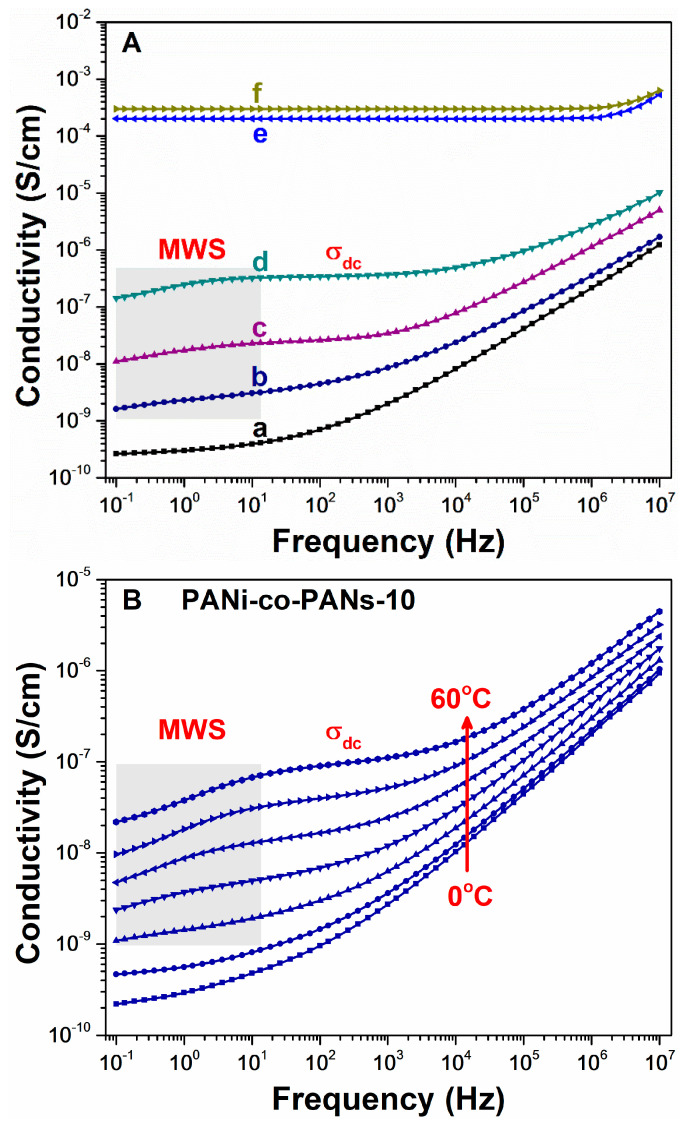
Evolution of electrical conductivity with electrical field frequency: (**A**) Comparative evolution of conductivity as a function of frequency for (a) PANs reference, (b) PANi-co-PANs-10, (c) PANi-co-PANs-20, (d) PANi-co-PANs-30, (e) PANi-co-PANs-40, and (f) PANi-co-PANs-60; (**B**) example of σ(f) dependence with temperature for PANi-co-PANs-10. In (**A**), the isothermal σ(ƒ) profiles are presented at room temperature (25 °C). The grey colored area indicates the effect of MWS process on the σ(ƒ) dependences.

The dc-conductivity of the homologous series of amphiphilic PANi-co-PANs was investigated with respect to the alternating electrical field frequency, temperature, and the increase of the PANi amount. The evolution of electrical conductivity with frequency σ(ƒ) is comparatively presented in Figure 5A for amphiphilic polyaniline copolymers and PANs, as a reference sample. For the isothermal σ(ƒ) dependency of the simple PANi sample, two different frequency regions were detected: (i) in the high frequency region, the linear decrease of conductivity was of capacitive-type, and may be assigned to the dipolar relaxation phenomena of the material, (ii) at low frequencies, the flat region is of resistive-type and generally attributed to the movement of free charge carriers through the material lattice (dc-conductivity) [37,38]. Following the σ(ƒ) profiles for the homologous series of PANi-co-PANs copolymers, with the increase in the PANi chain length, the plateau region of dc-conductivity is enlarged, while the capacitive regime is limited to higher frequencies. As expected, the magnitude of conductivity increased gradually within the homologous PANi-co-PANs series with the increase in the conductive PANi chain in the entire frequency range, reaching the maximum for the PANi-co-PANs-60. Interestingly, for the σ(ƒ) dependencies of the copolymers with a shorter PANi chain, as the frequency decreased, the flat region was followed by a progressive decrease in conductivity. According to the literature, heterogeneous materials consisting of two components with different conductivities (e.g., an insulating component and a conductive one) are frequently affected by the process of interfacial polarization. This phenomenon is well known as Maxwell-Wagner-Sillars (MWS) polarization and is caused by changes in the local conductivity that may appear across internal interfaces [38,39]. In the current case, MWS could have been caused by a high disparity in electrical conductivity between the PANi and PANs chains, which could lead to charge accumulation at the interface between the two, a phenomenon frequently observed in nanocomposites of highly conductive fillers in an insulating polymer matrix [40]. For example, in the case of copolymer PANi-co-PANs-10, the interfacial MWS signal increased at low frequencies and expanded to higher frequencies with increasing temperature (Figure 5B). The copolymers PANi-co-PANs-20 and PANi-co-PANs-30 revealed a similar behavior. Returning to the findings presented in Figure 5A, at room temperature, the MWS process directly intensified PANi chain length. However, the polarization signal was not highlighted in the σ(ƒ) profiles of PANi-co-PANs-40 and PANi-co-PANs-60 copolymers, because the PANi content was high and, consequently, the resistive behavior was dominant.

**Figure 6:**
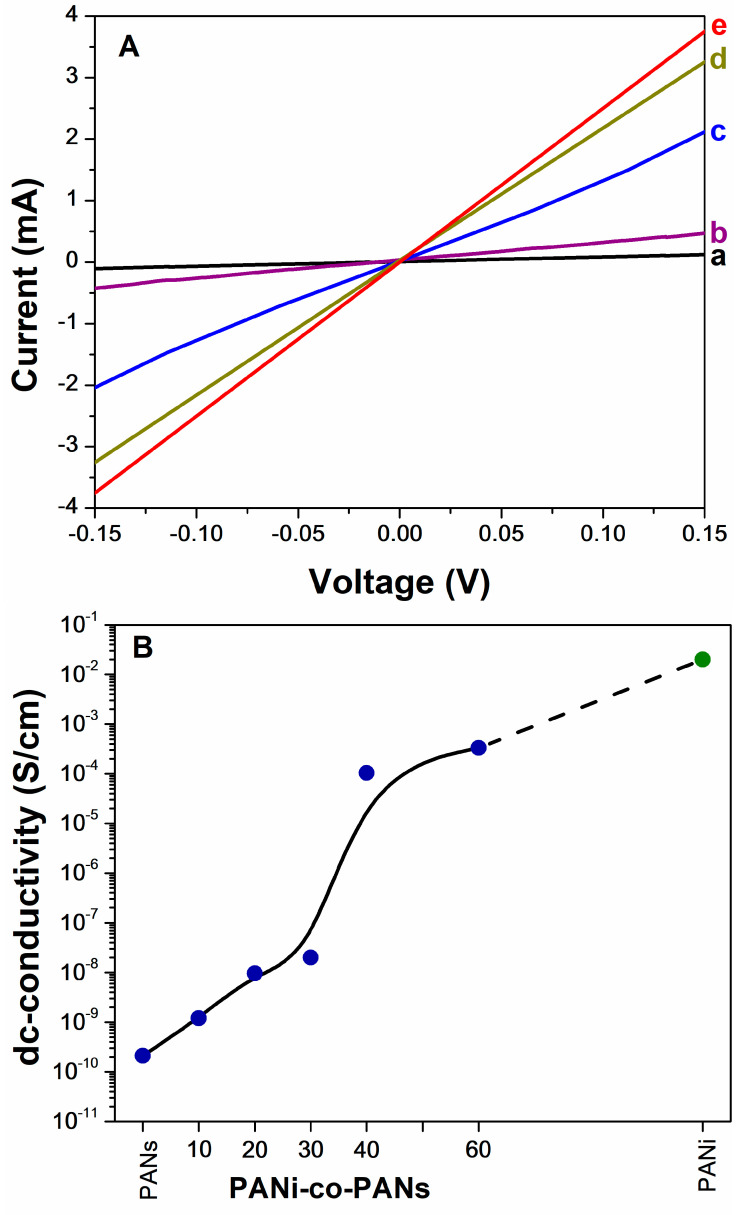
(**A**) The current–voltage curves for (a) PANs reference, (b) PANi-co-PANs-20, (c) PANi-co-PANs-40, (d) PANi-co-PANs-60, and (e) PANi; (**B**) evolution of dc-conductivity with increasing content of PANi component. The measurements were recorded at room temperature. In (**A**), for a better visualization of the I-V characteristics, the current values for PANs were multiplied by 10^5^, and those of the PANi-co-PANs-20 sample were multiplied 10^4^. The b-spline function in (**B**) is used as a guide to the eyes.

As previously observed, the electrical conductivity measured in the ac-regime is strongly affected by the dipolar relaxation and interfacial polarization. The latter processes are commonly retrieved in the dielectric-type spectra due to the imminent movement of dipoles, as well as electric charges with the oscillations of the external alternating electrical field. Therefore, the intrinsic electrical conductivity of the polyaniline derivative block copolymers was further evaluated in a dc-regime. In Figure 6A, the typical I-V characteristics of all measured samples are depicted as linear dependencies. The highest values of measured current were retrieved for PANi-co-PANs-40 and PANi-co-PANs-60, while that for copolymers with a lower content of PANi it was limited to between µA and nA. As follows from Figure 6B and Table S2, the σ_dc_ of the homologous series of polyaniline derivative block copolymers was gradually enhanced with the PANi length. We note that PANi-co-PANs-40 and PANi-co-PANs-60 presented σ_dc_ values around 10^−4^ S/cm, being in the range of semiconducting-type materials. For comparison, the dc-conductivity of PANi was measured with a standard four-point probe instrument, and the numerical value was found to be 2 × 10^−2^ S/cm.

**Figure 7:**
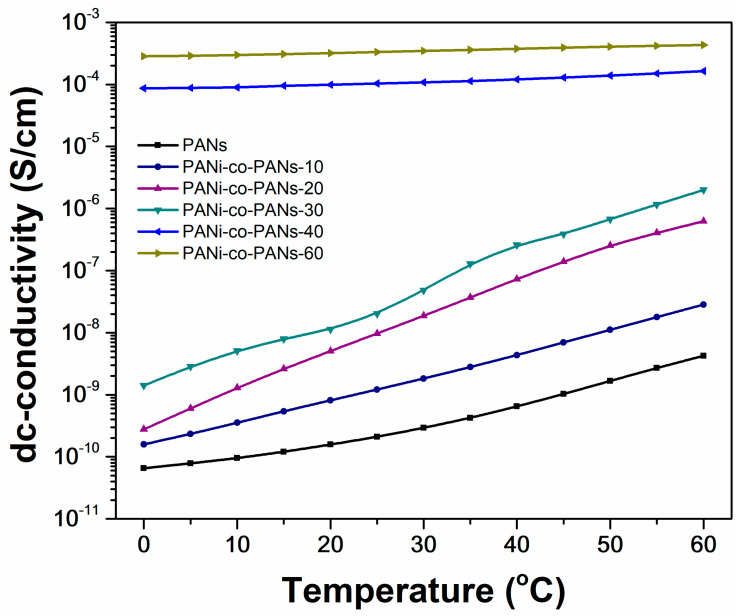
Evolution of dc-conductivity with temperature for polyaniline copolymers (PANi-co-PANs) and PANs reference.

Figure 7 shows the evolution of dc-conductivity at temperatures between 0 °C and 60 °C for block copolymers and PANs samples. The copolymers with a shorter length of PANi chain were the most sensitive to the temperature changes (e.g., for PANi-co-PANs-20, σ_dc_ is enhanced by one order of magnitude, between 10^−9^ S/cm to 10^−8^ S/cm when temperature increased between 10 °C and 30 °C) while the σ_dc_ of PANi-co-PANs-40 and PANi-co-PANs-60 was slightly changed (e.g., for PANi-co-PANs-60, at 0 °C σ_dc_ = 2.8 × 10^−4^ S/cm and at 60 °C σ_dc_ = 4.3 × 10^−4^ S/cm). We believe the reason for this is that although PANs and, consequently the PANs segment in the PANi-co-PANs copolymers, behaves more like a dielectric at low temperature, it becomes increasingly more conductive with the increase in temperature; within almost two orders of magnitude. This is a typical behavior for semiconducting polymer materials, which experience an increase in charge carrier density, holes, and electrons, in the valence and conduction bands, respectively.

## 4. Conclusions

We have demonstrated that the homologous series of PANi-co-PANs amphiphilic polymers exhibit a gradual change in solubility, interfacial activity, wettability, and conductivity, within the boundaries set by the electrically insulating, hydrophilic PANs and the highly conductive, hydrophobic PANi. Within this series PANi-co-PANs-30 exhibits the highest interfacial activity but a rather modest conductivity of only 2 × 10^−8^ S/cm. On the other hand, the conductivity values jump in the homologous series to a value of 1 × 10^−4^ S/cm for PANi-co-PANs-40, which is partially soluble in, DMSO, ethanol, acetone, and isopropanol, suggesting that the amphiphilic balance leans toward a more hydrophobic and highly conductive PANi chain. Therefore, we conclude that for aqueous systems, the best performing amphiphiles in the homologous series with a relatively high conductivity are found within the interval set by PANi-co-PANs-20 and PANi-co-PANs-40. This homologous series of semiconducting amphiphiles could find important uses, both in the typical application realm of surfactants, such as in emulsification of water-insoluble monomers to generate nanomaterials with a conductive surface and for biosensing, but also in the realm of organic electronics. Future studies will focus on applications and improving the water solubility and conductivity of the homologous series by tuning the synthesis conditions.

## Data Availability

Not applicable.

## Supplementary Materials

The following supporting information can be downloaded at: https://www.mdpi.com/article/10.3390/polym14112149/s1, Figure S1: Photographs showing partial solubility of PANi-co-PANs-40 in DMSO and Ethanol+Isopropanol (1:1); Figure S2: FTIR spectra of amphiphile polyanilines copolymers *PANi-co-PANs*, PANs, PANi and physical mixture PANi-PANs. Each spectrum was normalized by the highest intensity peak and offset. (a) PANs, (b) PANi-co-PANs-2, (c) PANi-co-PANs-10, (d) PANi-co-PANs-20, (e) PANi-co-PANs-30, (f) PANi-co-PANs-40, (g) PANi-co-PANs-60, (h) physical mixture PANi- PANs and (i) PANi; Figure S3: ^1^H-NMR spectra of amphiphile polyanilines copolymers PANi-co-PANs in D_2_O-(a) PANi-co-PANs-2, (b) PANi-co-PANs-10, (c) PANi-co-PANs-20, (d) PANi-co-PANs-30; Figure S4: Contact angles of water on various substrates; Table S1: The ratio of the integral of aliphatic signal to aromatic signal; Table S2: Numerical values of dc-conductivity retrieved from the I-V characteristics.
