# Supplementary material for: Homologous Series of Polyaniline Derivatives Block Copolymers with Amphiphilic and Semiconducting Properties
Source: Polymers (Basel). 2022 May 25;14(11):2149. doi: 10.3390/polym14112149 (PMC9182807; doi:10.3390/polym14112149)
Supplement: Supplementary file 1 [file polymers-14-02149-s001.zip › polymers-1704921-supplementary.pdf]

Supporting Information

Homologous Series of Polyaniline Derivatives Block Copolymers with Amphiphilic and Semiconducting Properties

Ana-Maria Solonaru, Asandulesa Mihai and Andrei Honciuc *

Electroactive Polymers and Plasmochemistry Laboratory , “Petru Poni” Institute of Macromolecular Chemistry, Aleea Gr. Ghica Voda 41A, 700487 Iasi, Romania; solonaru.anamaria@icmpp.ro (A.-M.S.); asandulesa.mihai@icmpp.ro (M.A.)

* Correspondence: honciuc.andrei@icmpp.ro

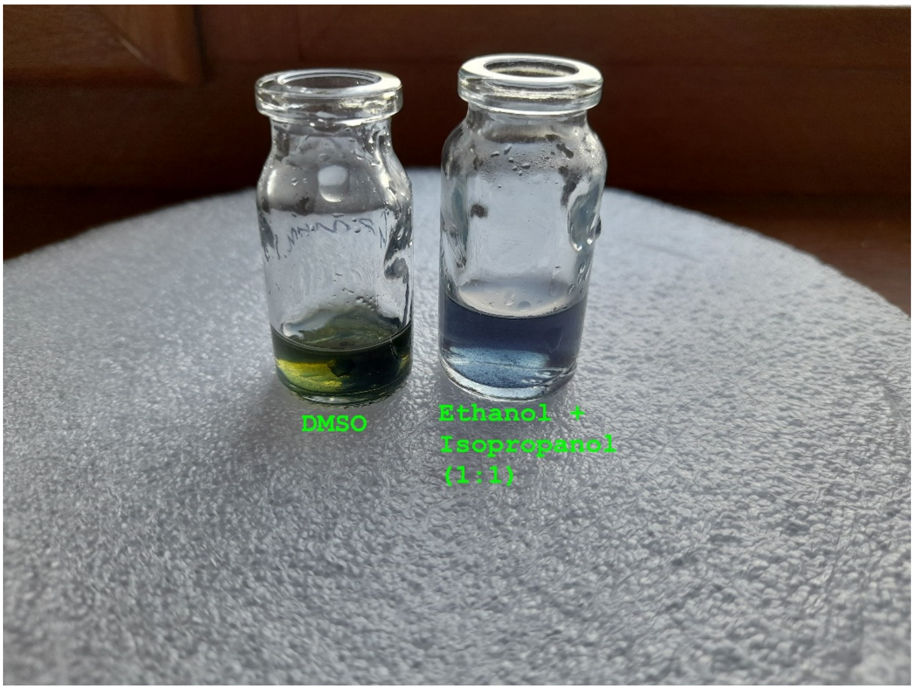

Figure S1. Photographs showing partial solubility of PANi-co-PANs-40 in DMSO and Ethanol+Isopropanol (1:1).

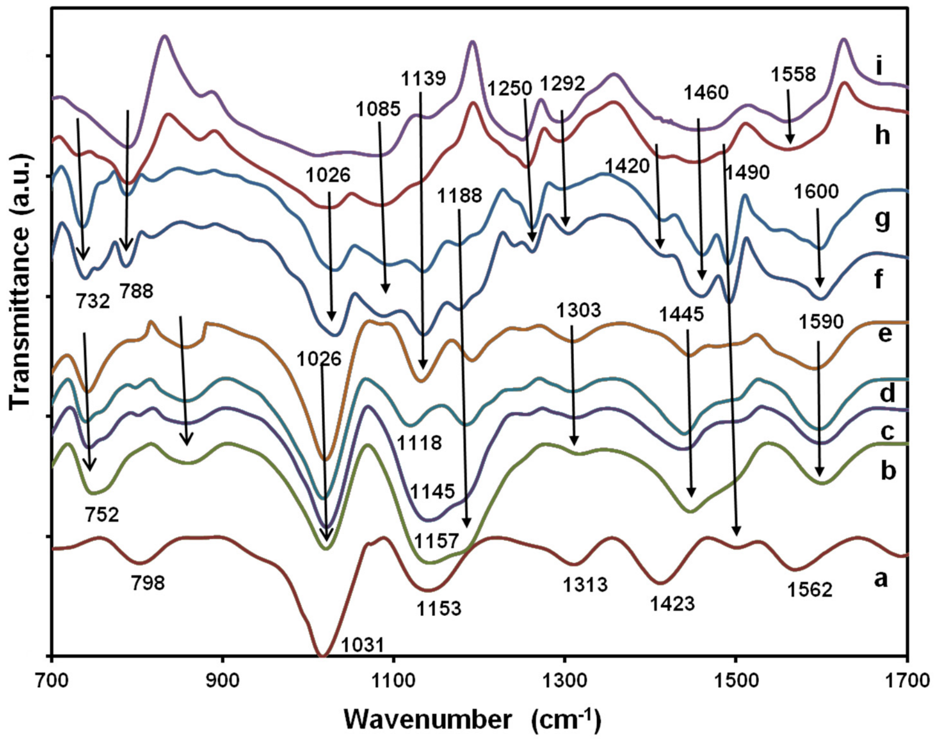

Figure S2. FTIR spectra of amphiphile polyanilines copolymers *PANi-co-PANs*, PANs, PANi and physical mixture PANi-PANs. Each spectrum was normalized by the highest intensity peak and offset .(a) PANs, (b) PANi-co-PANs-2, (c) PANi-co-PANs-10, (d) PANi-co-PANs-20,(e) PANi-co-PANs-30, (f) PANi-co-PANs-40, (g) PANi-co-PANs-60, (h) physical mixture PANi- PANs and (i) PANi

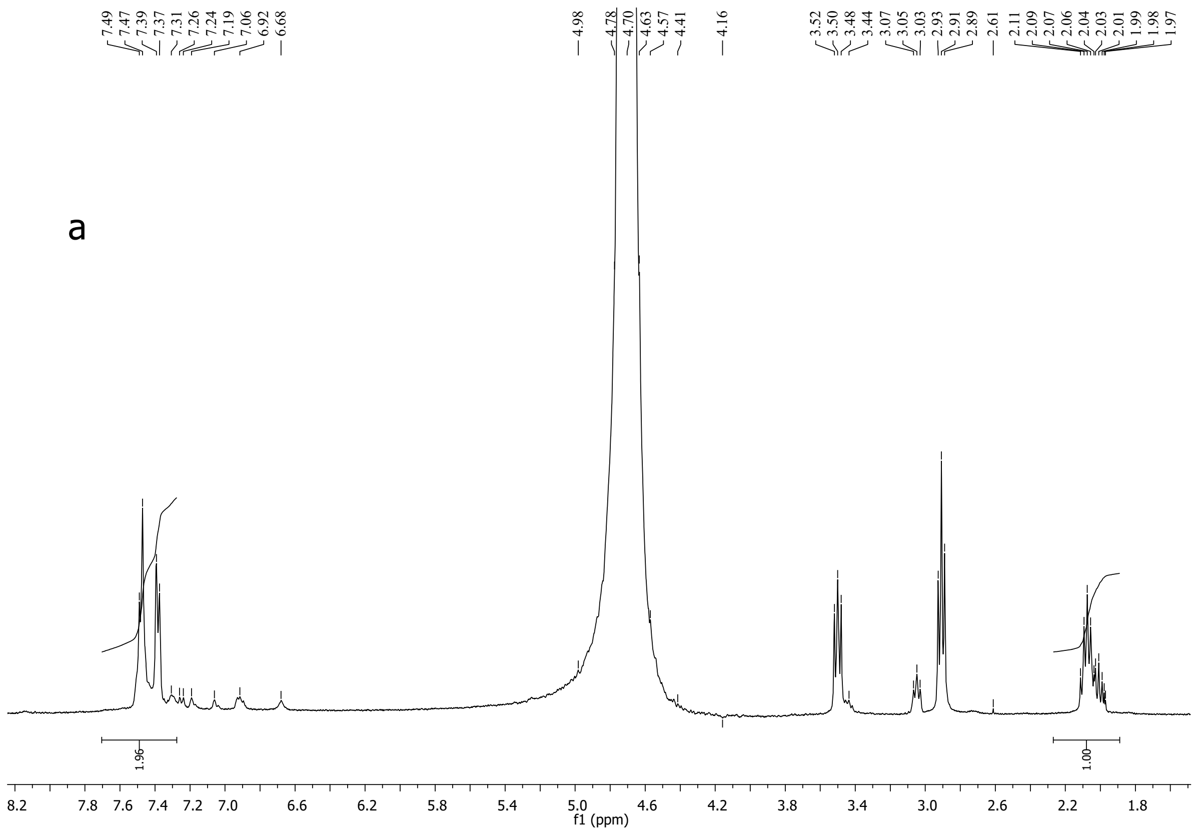
a
7.49 7.47 7.39 7.37 7.31 7.26 7.24 7.19 7.06 6.92 6.68
4.98 4.78 4.70 4.63 4.57 4.41 4.16
3.52 3.50 3.48 3.44 3.07 3.05 3.03 2.93 2.91 2.89 2.61
2.11 2.09 2.07 2.06 2.04 2.03 2.01 1.99 1.98 1.97
1.96
1.00
f1 (ppm)

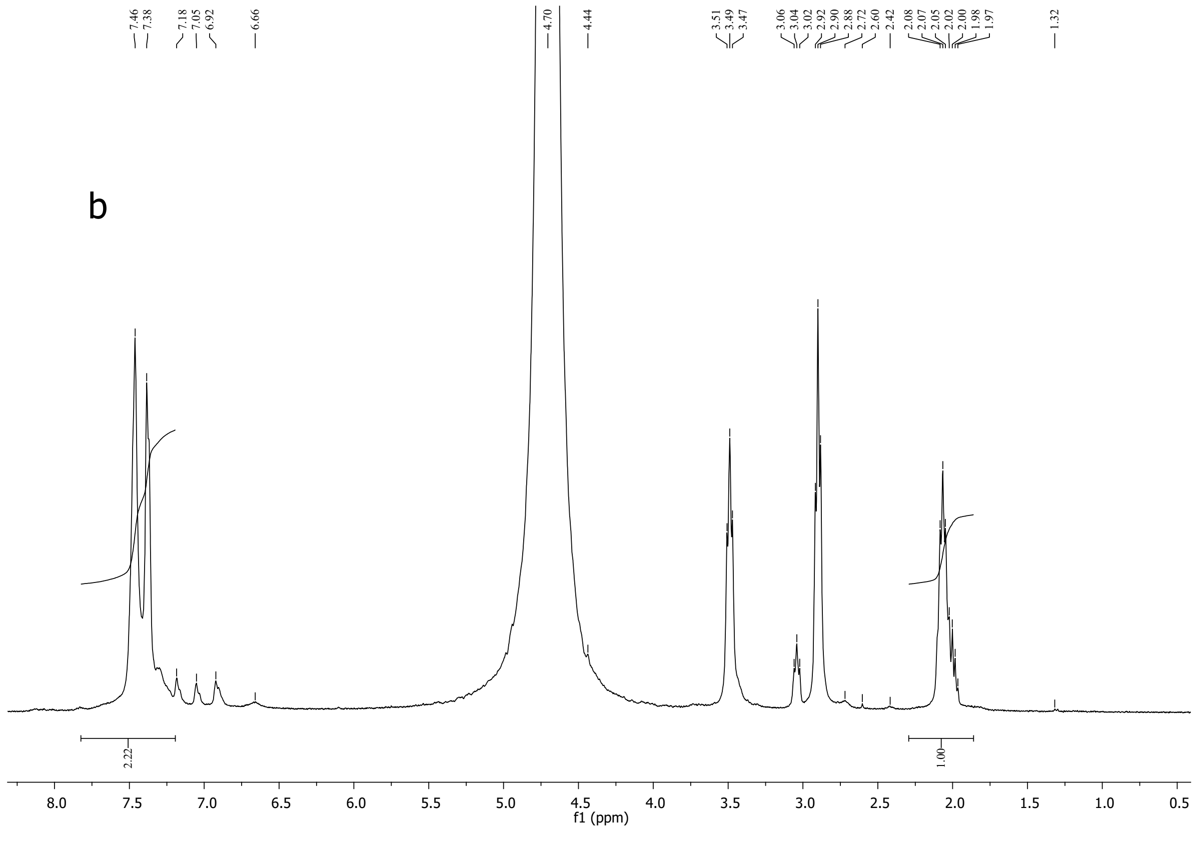
b
7.46 7.38 7.18 7.05 6.92 6.66
4.70 4.44
3.51 3.49 3.47 3.06 3.04 3.02 2.92 2.90 2.88 2.72 2.60 2.42
2.08 2.07 2.05 2.02 2.00 1.98 1.97 1.32
2.22
1.00
f1 (ppm)

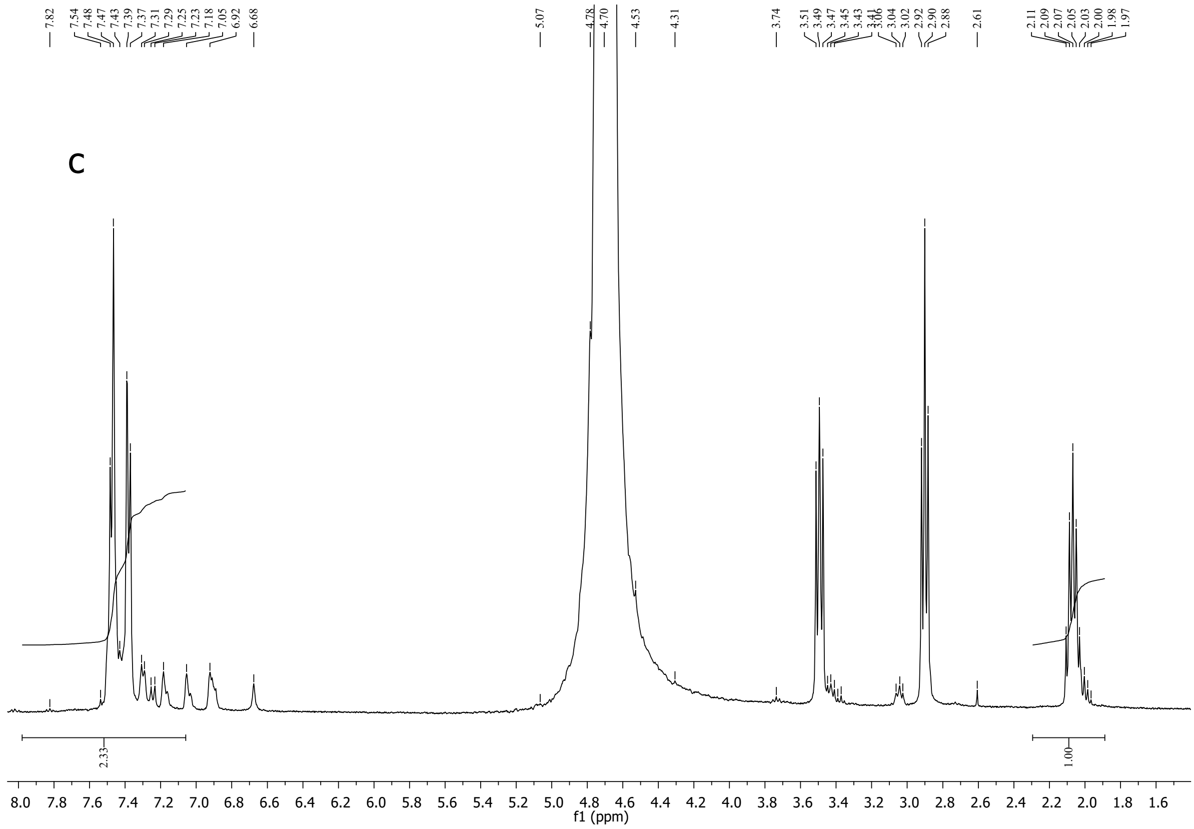
7.82
7.54
7.48
7.47
7.43
7.39
7.37
7.31
7.29
7.25
7.23
7.18
7.05
6.92
6.68
5.07
4.78
4.70
4.53
4.31
3.74
3.51
3.49
3.47
3.45
3.43
3.41
3.06
3.04
3.02
2.92
2.90
2.88
2.61
2.11
2.09
2.07
2.05
2.03
2.00
1.98
1.97
c
2.33
1.00
f1 (ppm)

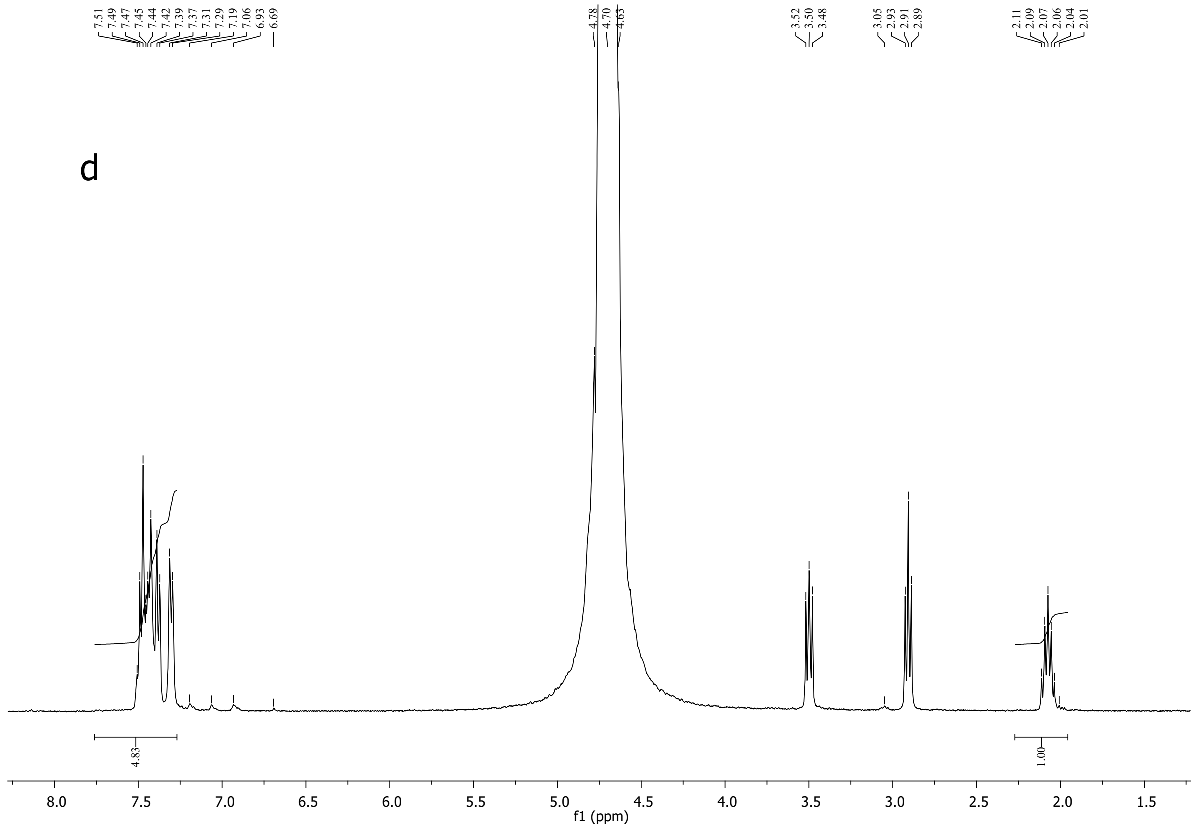
7.51
7.49
7.47
7.45
7.44
7.42
7.39
7.37
7.31
7.29
7.19
7.06
6.93
6.69
4.78
4.70
4.63
3.52
3.50
3.48
3.05
2.93
2.91
2.89
2.11
2.09
2.07
2.06
2.04
2.01
d
4.83
1.00
f1 (ppm)

Figure S3. ^{1}H-NMR spectra of amphiphile polyanilines copolymers PANi-co-PANs in D_2O- (a) PANi-co-PANs-2, (b) PANi-co-PANs-10, (c) PANi-co-PANs-20, (d) PANi-co-PANs-30.

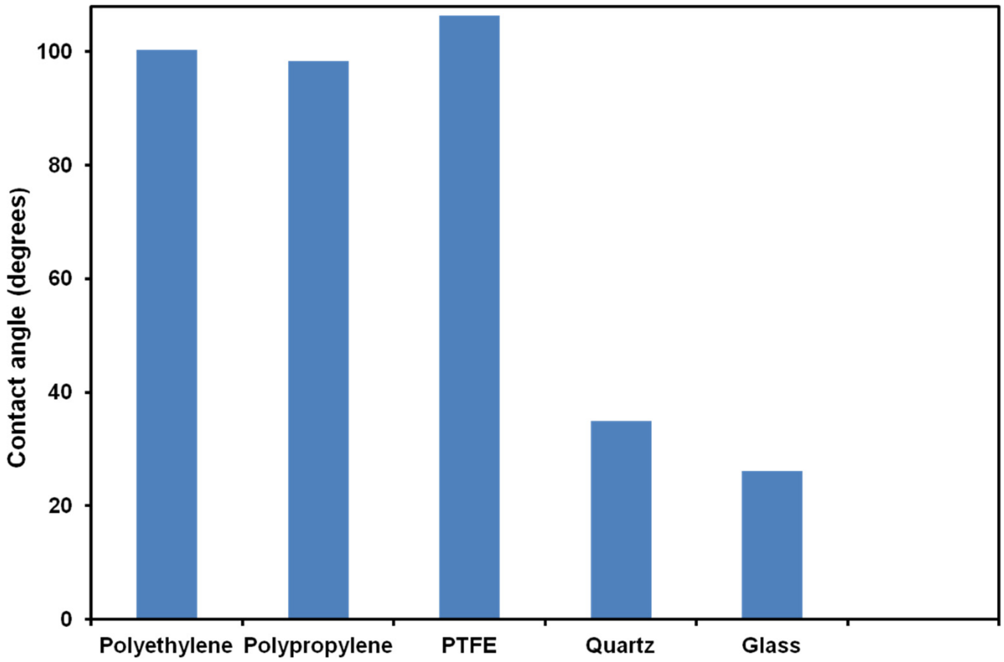

Figure S4. Contact angles of water on various substrates.

Table S1. The ratio of the integral of aliphatic signal to aromatic signal.

Copolymer	**Ratio $\int_{Aliphatic}/\int_{Aromatic}$**
PANi-co-PAN-2	1/1.96
PANi-co-PAN-10	1/2.22
PANi-co-PAN-20	1/2.33
PANi-co-PAN-30	1/4.83

Note: $\int_{Aliphatic}$—integral of the aliphatic signal and $\int_{Aromatic}$—integral of the aromatic signal.

Table S2. Numerical values of dc-conductivity retrieved from the I-V characteristics.

**Sample**	**dc-conductivity (S/cm)**
PANs	2×10^{-10}

PANi-co-PANs-10	1×10^{-9}
PANi-co-PANs-20	1×10^{-8}
PANi-co-PANs-30	2×10^{-8}
PANi-co-PANs-40	1×10^{-4}
PANi-co-PANs-60	3×10^{-4}
PANi	2×10^{-2}
